# The prognostic value of total T3 after acute cerebral infarction is age-dependent: a retrospective study on 768 patients

**DOI:** 10.1186/s12883-019-1264-z

**Published:** 2019-04-05

**Authors:** Lei-Qing Li, Xiao-Yan Xu, Wen-Yu Li, Xing-Yue Hu, Wen Lv

**Affiliations:** 1 Department of Intensive Care Unit, the Second Affiliated Hospital, Zhejiang University School of Medicine, Hangzhou, Zhejiang, 310009 China; 2 Department of Neurology, the Sir Run Run Shaw Hospital, Zhejiang University School of Medicine, Zhejiang, 310016 Hangzhou China

**Keywords:** Thyroid-related hormones, Ischemic stroke, Functional outcome

## Abstract

**Background:**

Serum triiodothyronine (T3) concentration was reported to be associated with the prognosis after acute ischemic stroke. The aim of this study was to evaluate the effect of age on the prognostic value of thyroid-related hormones after an acute ischemic stroke.

**Methods:**

This was a retrospective study involving the review of 1072 ischemic stroke patients who had been consecutively admitted to the hospital within 72 h of symptom onset. Total triiodothyronine (T3), total thyroxine (T4), free T3, free T4, and thyroid-stimulating hormone (TSH) were assessed to determine their values for predicting functional outcome at the first follow-up clinic visits, which usually occurred 2 to 4 weeks after discharge from the hospital.

**Results:**

A total of 768 patients were finally included in the study and divided into two age groups: a younger group (age < 65 years) and an older group (age ≥ 65 years). On univariate analysis, four factors—lower total T3, free T3 concentrations, higher scores on the National Institute of Health Stroke Scale (NIHSS) and the presence of atrial fibrillation—were associated with poor functional outcomes in both groups. In addition, older age, female gender, higher free T4, and lower TSH levels were also associated with poor function in the older group. On multiple logistic regression analysis, higher NIHSS scores (odds ratio [OR] =1.95; 95% confidence interval [CI], 1.66–2.30; *P* ≤ .001) and lower total T3 concentrations (OR = 0.06; 95% CI, 0.01–0.68; *P* = .024) remained independently associated with poor functional outcome in the older group. However, the independent association with poor function of lower total T3 was not confirmed in the younger group.

**Conclusions:**

The prognostic value of low total T3 is age-associated and more meaningful in an older population.

## Background

Previous studies have shown an association of serum triiodothyronine (T3) concentration with prognosis after acute ischemic stroke; that is, a reduction in T3 level without an elevation in thyroid-stimulating hormone (TSH) (i.e., low T3 syndrome) was reported to be associated with poorer clinical outcomes [1–4]. Better survival after acute stroke was found in one study [1] even when the cut-off point used was the median T3 level, showing that a lower T3 level, even when within the normal range, may be associated with a poorer prognosis. Furthermore, our previous study confirmed that a lower total T3 concentration within the normal range was independently associated with a poorer short-term outcome after ischemic stroke [5].

As is well known, an individual’s levels of thyroid hormones change with age: serum total and free T3 levels are reported to decrease with age [6, 7], whereas serum total and free thyroxine (T4) levels remain unchanged [6–9] or increase [10]. It is further believed that these alterations create major difficulties in attempting to determine thyroid status in the elderly with precision [11]. As a special physiologic status, aging may also influence the prognostic value of some known or possible predictors of outcome after stroke; Therefore, this study was designed to investigate the effect of age on the prognostic value of thyroid-related hormones after acute ischemic stroke.

## Methods

### Study population

We reviewed all of the available charts of patients admitted with acute ischemic stroke at the acute stroke unit of the Sir Run Run Shaw Hospital, Zhejiang University School of Medicine, between January 2011 and December 2013. Included patients met the following criteria: (1) a diagnosis of acute ischemic stroke with a new focal neurologic deficit and a corresponding lesion on magnetic resonance imaging or delayed computed tomography; on admission, non-contrast head computed tomography scan was perfomed to exclude intracranial hemorrhage; (2) admission within 72 h after symptom onset; and (3) thyroid function testing within 24 h of admission. Patients with known thyroid disease, biochemically defined overt thyroid disease, or the use of medications that affect thyroid function were excluded. To avoid any confounding effects, subjects with cancer, hematologic diseases, severe renal or liver failure, and inflammatory or infectious diseases were also excluded. All patients received standard routine treatment and intravenous thrombolysis was considered for all patients presenting within 4.5 h of symptom onset.

### Data collection

Baseline characteristics—including demographic data; stroke risk factors, such as a history of hypertension, smoking, diabetes mellitus, hyperlipidemia, transient ischemic attacks, coronary artery disease, and atrial fibrillation; laboratory parameters routinely measured in the emergency department, including routine blood count, admission glucose, and C-reactive protein (CRP) levels; and neurologic deficits—were recorded. Blood samples for CRP were taken at the time of admission and analyzed by an immunoturbidimetric method using the Abbott Architect c16000 Biochemistry Analyzer (Abbott Laboratories Inc., LakeBluff, IL, USA).

Thyroid function tests examined TSH, total T3, total T4, free T3, and free T4 levels. Serum concentrations of these hormones were measured using the Abbott i4000 Chemiluminescent Microparticle Immunoassay (Abbott Laboratories Inc., LakeBluff, IL, USA). Normal ranges of thyroid hormones are as follows: TSH, 0.35–4.94 IU/L; total T3, 0.57–1.59 ng/mL; total T4, 4.87–11.72 μg/dL; free T3, 1.71–3.71 pg/mL; and free T4, 0.7–1.48 ng/dL.

The severity of stroke on admission was assessed using the National Institutes of Health Stroke Scale (NIHSS). Stroke subtype was divided into 3 groups according to the vessels involved: anterior circulation group, posterior circulation group, and both anterior and posterior circulation group. Functional outcomes were evaluated using the modified Rankin Scale (mRS) at the first follow-up clinic visit, which usually occurred 2 to 4 weeks after discharge from the hospital. Patients were classified into 2 outcome groups: poor functional outcome (mRS > 2) and good functional outcome (mRS ≤2). All of the evaluations were performed by the study neurologists.

### Data analysis

Data were analyzed using SPSS 22.0 software for Windows. Data are expressed as medians (interquartile range [IQR]) or mean ± standard deviation (SD) for quantitative variables and as numbers (percent) for qualitative variables. Pearson correlation was used to evaluate the association of age with thyroid hormone levels. The differences in thyroid-related hormones were evaluated with the division of data into two groups based on age (< 65 years for the younger group and ≥ 65 years for the older group) [12, 13]. Simultaneous comparisons of other parameters in the two age-based groups were performed. In addition, comparisons with clinical and laboratory variables between different outcome groups were determined in each group. Continuous data were compared by independent sample *t* tests and, for categorical variables, chi-square tests. Besides, spearman’s rank correlation was performed to assess the association between thyroid hormone levels and stroke severity on admission in each group. Then, the variables associated with significant differences between outcomes in each of the two groups were included in multiple logistic regression analysis and a backward likelihood ratio test was used to identify the independent predictors of poor functional outcome after stroke in each group. Receiver operating characteristics (ROC) curve analysis was used to assess discrimination and results are reported as area under the curve (AUC). All of the statistical tests were two-tailed, and *P* < .05 indicated statistical significance.

## Results

During the study period, 1072 ischemic stroke patients were admitted to our acute stroke unit within 72 h after symptom onset. Of these, 768 patients were included in the finally analysis and 304 patients were excluded. The reasons for exclusion were as follows: previous thyroid disease (*n* = 19); biochemically defined overt or subclinical thyroid disease (*n* = 57), defined as any of the following thyroid function tests alteration at admission: abnormal TSH, abnormal free T4 or total T4, raised free T3 or total T3 (*n* = 68, low free T3 or total T3 with normal TSH was taken as a marker of low T3 syndrome and therefore not considered as a primary thyroid disorder [14]); cancer (*n* = 51); hematologic diseases (*n* = 13); severe renal or liver failure (*n* = 26); inflammatory or infectious diseases (*n* = 32); and no thyroid function test within 24 h of admission (*n* = 38).

The baseline characteristics of the included patients are summarized in Table 1. Both the younger and older groups had a male predominance, however, the proportion of males appeared to decrease with age (57.6% in older group versus 66.4% in younger group, *P* = .016). There was no significant difference in admission NIHSS (*P* = .703) and artery system involved between the two groups. Analysis of parameters related to patient history between the two groups showed that the older group included more patients with a history of previous cerebral infarction (*P* ≤ .001), atrial fibrillation (*P* ≤ .001), and hypertension (*P* = .001), while the younger group included more patients with a history of hyperlipidemia (*P* ≤ .001) and smoking (*P* ≤ .001). In addition, compared with the patients in the younger group, the subjects in the older group had higher levels of blood pressure on admission (*P* ≤ .001 for both systolic and diastolic blood pressure), with higher TSH (*P* = .002), free T4 (*P* = .011) and CRP levels (*P* = .001) and lower total T3 and free T3 concentrations (both *P* ≤ .001).

**Table 1:** Baseline characteristics of patients with acute ischemic stroke (grouped by age)

Variables	Younger Age Group (age < 65 yr) (*n* = 327)	Older Age Group (age ≥ 65 yr) (*n* = 441)	*P* Value
Age (years) (median, IQR)	57 (51–61)	76 (70–80)	≤.001
Sex (male, %)	217 (66.4%)	254 (57.6%)	.016
Smoking (n,%)	167 (51.1%)	148 (33.6%)	≤.001
Hyperlipidemia (n,%)	177 (54.1%)	128 (29.0%)	≤.001
Coronary artery disease (n,%)	21 (6.4%)	39 (8.8%)	.225
Diabetes mellitus (n, %)	84 (25.7%)	104 (23.6%)	.553
Atrial fibrillation (n, %)	16 (4.9%)	56 (12.7%)	≤.001
Previous stroke (n, %)	21 (6.4%)	69 (15.6%)	≤.001
Transient ischemic attacks (n, %)	9 (2.8%)	15 (3.4%)	.679
History of hypertension (n, %)	191 (58.4%)	311 (70.5%)	.001
Systolic blood pressure (mmHg) (median, IQR)	152 (132–170)	160 (142–175)	≤.001
Diastolic blood pressure (mmHg) (median, IQR)	85 (77–95)	80 (71–88)	≤.001
Baseline NIHSS scores (median, IQR)	2 (1–5)	2 (1–5)	.703
Anterior circulation group	229 (70.0%)	304 (68.9%)	.752
Posterior circulation group	91 (27.8%)	125 (28.3%)	.935
TSH (IU/L) (median, IQR)	1.52 (1.01–2.02)	1.67 (1.05–2.40)	.002
total T4(μg/dL) (median, IQR)	6.41 (5.58–7.27)	6.59 (5.82–7.50)	.066
total T3 (ng/mL) (median, IQR)	0.85 (0.72–0.98)	0.81 (0.69–0.92)	≤.001
free T4 (ng/dL) (median, IQR)	1.07 (0.99–1.17)	1.11 (1.00–1.21)	.011
free T3 (pg/mL) (median, IQR)	2.54 (2.29–2.89)	2.45 (2.16–2.74)	≤.001
Initial WBC count (× 10^9^/L) (median, IQR)	7.20 (5.90–9.45)	6.80 (5.60–8.30)	.051
Glucose on admission (mmol/L) (median, IQR)	7.07 (5.82–9.59)	7.09 (5.82–9.46)	.471
C-reactive protein (mg/dL) (median, IQR)	1.40 (0.80–2.70)	2.40 (0.90–5.98)	.001
poor functional outcome (mRS > 2) (n, %)	38 (11.6%)	64 (14.5%)	.282

*IQR* interquartile range, *NIHSS* National Institutes of Health Stroke Scale, *TSH* thyroid-stimulating hormone, *T4* thyroxine, *T3* triiodothyronine, *WBC* white blood cell, *mRS* modified Rankin Scale

There were negative correlations between age and levels of total T3 (*r* = − 0.190, *P* ≤ .001) and free T3 (*r* = − 0.203, *P* ≤ .001) and positive correlations between age and levels of total T4 (*r* = 0.077, *P* = .032) and free T4 (*r* = 0.115, *P* = .001) but not TSH (*P* = .058).

There were negative correlations between NIHSS scores and levels of total T3 (*r* = − 0.159, *P* = 0.004 in the younger group; *r* = − 0.197, *P* = 0.000 in the older group) and free T3 (*r* = − 0.186, *P* = 0.001 in the younger group; *r* = − 0.232, *P* = 0.000 in the older group) in both the younger and older groups. Besides, there were positive correlations between NIHSS scores and levels of total T4 (*r* = 0.112, *P* = 0.042) in the younger group, while a negative correlation was found between NIHSS scores and TSH (*r* = − 0.095, *P* = 0.046) in the older group.

At the study’s end point, a total of 38 patients (11.6%) in the younger group had poor functional outcomes with no significant difference as compared with the older group (64 subjects, 14.5%, *P* = .282). As shown in Table 2, the variables associated with poor functional outcome on univariate analysis in the two groups were not the same. In the younger group, poor functional outcome on univariate analysis was associated with lower total T3 and free T3 concentrations, higher NIHSS scores and the presence of atrial fibrillation. In the older group, except the four items mentioned in the younger group, older age, female gender, higher free T4, and lower TSH levels were also associated with poor function on univariate analysis.

**Table 2:** Clinical and laboratory findings on admission according to outcome in the two age groups

Variables	Younger Group (age < 65 yr)		*P* Value	Older Group (age ≥ 65 yr)		*P* Value
	Good functional outcome (mRS ≤ 2) (*n* = 289)	Poor functional outcome (mRS > 2) (*n* = 38)		Good functional outcome (mRS ≤2) (*n* = 377)	Poor functional outcome (mRS > 2) (*n* = 64)	
Age (years)^a^	57 (51–61)	58 (52–62)	.169	75 (70–80)	78 (72–83)	.016
Sex (male, %)	191 (66.1%)	26 (68.4%)	.856	225 (59.7%)	29 (45.3%)	.040
Smoking (n,%)	142 (49.1%)	25 (65.8%)	.054	133 (35.3%)	15 (23.4%)	.064
Hyperlipidemia (n,%)	156 (54.0%)	21 (55.3%)	.881	105 (27.9%)	23 (40.0%)	.188
Coronary artery disease (n, %)	16 (5.5%)	5 (13.2%)	.081	33 (8.8%)	6 (9.4%)	.871
Diabetes mellitus (n, %)	75 (26.0%)	9 (23.7%)	.846	87 (23.1%)	17 (26.6%)	.633
Atrial fibrillation (n, %)	10 (3.5%)	6 (15.8%)	.006	41 (10.9%)	15 (23.4%)	.008
Previous stroke (n, %)	18 (6.2%)	3 (7.9%)	.722	56 (14.9%)	13 (20.3%)	.352
Transient ischemic attacks (n, %)	7 (2.4%)	2 (5.3%)	.281	13 (3.4%)	2 (3.1%)	.625
History of hypertension (n, %)	168 (58.1%)	23 (60.5%)	.862	270 (71.6%)	41 (64.1%)	.237
Systolic blood pressure (mm Hg)^a^	152 (131–170)	151 (135–165)	.525	161 (141–176)	158 (147–171)	.469
Diastolic blood pressure (mm Hg)^a^	85 (77–95)	83 (78–99)	.610	80 (71–88)	77 (67–87)	.276
Baseline NIHSS scores^a^	2 (1–3)	11 (8–15)	≤.001	2 (1–4)	11 (8–14)	≤.001
TSH (IU/L)^a^	1.52 (1.01–2.05)	1.60 (1.14–1.96)	.951	1.73 (1.09–2.45)	1.36 (0.85–1.98)	.021
total T4 (μg/dL)^a^	6.37 (5.55–7.23)	6.88 (5.89–7.82)	.068	6.51 (5.79–7.47)	6.96 (6.03–7.95)	.051
total T3 (ng/mL)^a^	0.86 (0.73–0.98)	0.76 (0.63–0.92)	.013	0.82 (0.71–0.93)	0.71 (0.57–0.83)	≤.001
free T4 (ng/dL)^a^	1.07 (0.98–1.17)	1.11 (0.99–1.21)	.257	1.10 (1.00–1.20)	1.13 (1.03–1.29)	.040
free T3 (pg/mL)^a^	2.59 (2.31–2.93)	2.40 (2.01–2.66)	.001	2.49 (2.20–2.77)	2.21 (1.92–2.53)	≤.001
Initial WBC count (×10^9^/L)^a^	7.10 (5.90–8.95)	8.60 (6.28–10.60)	.134	6.60 (5.60–8.30)	7.80 (6.30–8.40)	.175
Glucose on admission (mmol/L)^a^	7 (5.78–9.24)	8.19 (6.06–13.36)	.072	7.04 (5.81–9.36)	7.29 (5.85–10.72)	.516
C–reactive protein (mg/dL)^a^	1.40 (0.80–2.80)	1.7 (0.80–2.40)	.366	2.10 (0.90–5.90)	3.45 (1.25–8.13)	.876

*IQR* interquartile range, *NIHSS* National Institutes of Health Stroke Scale, *TSH* thyroid–stimulating hormone, *T4* thyroxine, *T3* triiodothyronine, *WBC* white blood cell, *mRS* modified Rankin Scale

^a^Expressed as median (IQR)

On multiple logistic regression analysis, only the NIHSS scores remained independently associated with poor functional outcome in the younger group (OR = 1.56; 95% CI 1.39–1.76; *P* ≤ .001). Neither total T3 nor free T3 was independently associated with a poor functional outcome in this group. However, in the older group, in addition to the NIHSS scores (OR = 1.95; 95% CI 1.66–2.30; *P* ≤ .001), lower total T3 (OR = 0.06; 95% CI 0.01–0.68; *P* = .024) was also confirmed to be independently associated with poor functional outcome, even though with a less AUC [0.66 (95% CI 0.59–0.74) than NIHSS [0.87(95% CI 0.85–0.88)]. This finding indicates that the prognostic value of a lower total T3 is age-associated and more meaningful in an older population.

## Discussion

This study demonstrates that in euthyroid patients with acute ischemic stroke, the association of total T3 level with functional outcome differs with age. In patients at or above 65 years of age, a lower total T3 concentration was an independent predictor of poor functional outcome after ischemic stroke. However, none of the thyroid–related hormones, including total T3, were independently associated with a poor functional outcome in patients below 65 years of age.

Low T3 syndrome and lower T3 serum concentrations are common complications in acute cerebrovascular disease [15] and are reported to be associated with greater mortality and poorer clinical outcomes after ischemic stroke [1–4, 15–17]. For example, it has been reported that ischemic stroke patients with low T3 syndrome were at increased risk for poor functional outcome at follow–up (scheduled from 2 to 4 weeks after ischemic stroke) [3]. In addition, better survival after acute stroke was found even when the cutoff was the median total T3 level [1]. Furthermore, our previous study showed that lower total T3 concentrations that were within the normal ranges were independently associated with poorer short-term outcomes after ischemic stroke [5]. Moreover, a meta-analysis conducted recently to evaluate the prognostic value of thyroid hormones in acute ischemic stroke confirmed the predictive value of lower T3 levels for a poor prognosis in acute ischemic stroke [18]. However, the present study shows that the prognostic value of lower total T3 for poor outcome after ischemic stroke may not be confirmed in patients of all ages. In those below age 65, neither total T3 nor free T3 concentrations were shown to be independently associated with functional outcome, suggesting that this association with outcome after cerebral infarction is age-dependent, having greater clinical significance in older patients. To the best of our knowledge, such an association has not been reported previously.

T3 has been shown to have neuroprotective effects in animal models. Several attempts have been made to directly investigate the neuroprotective actions of T3 in mice models of transient middle cerebral artery occlusion [19–21]. In these studies, the administration of T3 was associated with reduced infarct volume and a reduced neurologic deficit. The age association of T3 and prognosis after cerebral infarction in our study agrees with current knowledge regarding the physiologic changes in T3 levels and their regulation that occur with age. Serum total and free T3 levels have been found to be decreased [6, 7] in healthy older individuals. The same reduction in T3 with age was also found in our population. As for serum T4, some authors have found it to remain unchanged [6–9] whereas others have shown it to increase with age [10]. In our population, serum total and free T4 levels were demonstrated to be higher in older patients. These changes in thyroid-related hormone concentrations may be explained by a decrease in the peripheral conversion of T4 to T3, which may be due to the decrease activity of liver type I deiodinase with aging [6]. Therefore, compared with younger patients with similar T4 levels, older patients may have lower T3 concentrations, making the detrimental effect of lower T3 on disease prognosis more obvious.

On the other hand, it is well known that, within a few hours after the onset of stroke, plasma T3 decreases, and the magnitude of the change is related to the stroke severity [22–25]. The association between lower T3 concentrations and greater stroke severity was confirmed in another recent meta-analysis [26]. Our study also showed the negative association between T3 (both total and free) concentrations and stroke severity in both age groups. Therefore, some authors believed the lower T3 levels found in stroke patients with the worse prognosis probably represent a marker of the severity of the disease [1]. However, the lower T3 and stroke severity association was reduced in older age [26], and our study found that the association between lower total T3 levels and poor outcome remained after adjusting for NIHSS scores, age and other variables associated with poor function on univariate analysis in patients at or above 65 years of age. In this context, our study has demonstrated that lower total T3 levels predict poor outcome after stroke beyond disease severity in older population.

Ischemic stroke increasing in incidence with age and tends to present with more severe symptoms; it also has worse outcomes in the elderly compared with younger patients [27]. As a special physiologic status, aging may also influence the prognostic value of some known or possible predictors of outcome after stroke (i.e., lower total T3 levels), as shown in this study. Therefore, it was appropriate to choose a population with variation in patients’ ages in order to study the prognostic value of candidate predictors. However, much remains to be learned about the association between the thyroid hormone profile and stroke outcome prior to considering the evaluation of serum T3 concentrations in the routine clinical setting for risk stratification and prognostication in patients with acute ischemic stroke.

Several limitations should be considered in the interpretation of our study. First, this is a retrospective study, which poses the risk of selection bias. Second, we lacked long-term clinical follow-up data on our patients. Third, we only use a mRS cut-off value to evaluate the functional outcome after stroke. One previous study has shown that reduced T3 concentrations can serve as an independent biomarker of worse cognitive outcomes after ischemic stroke, a common complication in stroke patients [28]. Further studies including more patient-centered outcomes are needed. Another limitation is that we lack the data about ischemic stroke etiology, which may be helpful to assigned obtained results to the stroke etiology. Finally, we had only a single baseline measurement and no dynamic monitoring of thyroid function. It has been reported that T3 values remain low until poststroke day 5 and then recover by days 7 to 9 after acute hemispheric stroke [29]. Future studies comprising the dynamic evaluation of thyroid function after stroke are desirable for validating the predictive role of thyroid hormone levels in acute stroke.

## Conclusion

This study assessed the effect of age on the prognostic value of thyroid-related hormones in patients with acute ischemic stroke. We found that the prognostic value of lower total T3 for poor outcome after ischemic stroke may not always be confirmed in all age populations and that, instead, it may be more clinically meaningful in older patients. Therefore, selecting a population of the appropriate age in order to study the prognostic value of candidate predictors is reasonable.

## Abbreviations

AUC: Area under the curve
CI: Confidence interval
CRP: C-reactive protein
IQR: Interquartile range
mRS: Modified Rankin Scale
NIHSS: National Institute of Health Stroke Scale
OR: Odds ratio
ROC: Receiver operating characteristics
SD: Standard deviation
T3: Triiodothyronine
T4: Thyroxine
TSH: Thyroid-stimulating hormone
WBC: White blood cell
